# Draft Genome Sequence of Petroleum Oil-Degrading Marine Bacterium *Pseudomonas taeanensis* Strain MS-3, Isolated from a Crude Oil-Contaminated Seashore

**DOI:** 10.1128/genomeA.00818-13

**Published:** 2014-01-09

**Authors:** Sang-Yeop Lee, Seon Hee Kim, Dong-Gi Lee, Seyeon Shin, Sung Ho Yun, Chi-Won Choi, Young-Ho Chung, Jong Soon Choi, Hyung-Yeel Kahng, Seung Il Kim

**Affiliations:** Korea Basic Science Institute, Daejeon, Republic of Koreaa; Department of Environmental Education, Sunchon National University, Suncheon, Republic of Koreab

## Abstract

*Pseudomonas taeanensis* MS-3^T^, isolated from a crude oil-contaminated seashore in South Korea, is capable of degrading petroleum oils, such as gasoline, diesel, and kerosene. Here, we report the draft genome sequence of this strain, which consists of 5,477,045 bp, with a G+C content of 60.72%.

## GENOME ANNOUNCEMENT

Many *Pseudomonas* strains not only have been reported to degrade a variety of man-made pollutants, including petroleum oil compounds (1, 2), but have been successfully applied in the bioremediation of contaminated sites (3–5).

Recently, frequent severe contamination of the marine environment by oil spill accidents stimulated environmental microbiologists to isolate powerful oil-degrading bacteria that can be used for bioremediation of the marine environment (6). Microbial genomes and genes involved with oil biodegradation in the marine system have been also reported by a few microbiologists (7, 8).

*Pseudomonas taeanensis* MS-3^T^, isolated from crude oil-contaminated seashore in Taean, South Korea, was reported as a novel species of *Pseudomonas* capable of degrading petroleum oils, such as gasoline, diesel, and kerosene (9). *P. taeanensis* MS-3^T^ degraded approximately 80% of gasoline and 100% of kerosene and diesel, all of which were used at 3% concentration during 7 days of incubation under aerobic conditions.

Notably, the alkane hydroxylase gene in *P. taeanensis* MS-3^T^ shares 87.0%, 85.3%, and 82.1% similarity, based on 184 amino acids, with alkane 1-monooxygenases of *Pseudomonas aeruginosa* M18, *Pseudomonas mendocina* NK-01, and *Pseudomonas denitrificans* ATCC 13867, respectively. The availability of only limited information on *P. taeanensis* MS-3^T^ regarding these noble alkane genes responsible for the degradation of petroleum oils motivated our efforts to sequence the *P. taeanensis* MS-3^T^ genome.

The *P. taeanensis* MS-3^T^ draft genome was generated using the Illumina MiSeq platform (San Diego, CA). As the result of a paired-end library, we had 4,330,258 reads with a read length of 150 bp and a mate-paired library with an insert size of 5 kb. We obtained total 26,854,288 reads with a read length of 37 bp. The total amount of reads data is 5,056 Mb.

The sequencing data were assembled with CLC Genomics Workbench (CLC bio, Aarhus, Denmark) version 6.0.2 after trimming of data quality control by using PrinSeq-lite version 0.20.3 (10). All reads were assembled into 19 contigs. The total contig length is 5,477,045 bp, with a G+C content of 60.72% and an N_50_ contig length of 1,013,530 bp. All contigs generated were submitted to the Rapid Annotations using Subsystems Technology (RAST) server for genome annotation (11). As a result, the numbers of genes and protein coding sequences are predicted to be 5,111 and 5,058, respectively. Additionally, 3 rRNAs and 50 tRNAs were predicted. The strain is available at the Korean Collection for Type Culture (KCTC) and Japan Collection of Microorganisms (JCM).

### Nucleotide sequence accession numbers.

The draft genome sequence of *P. taeanensis* MS-3^T^ has been deposited at DBBJ/EMBL/GenBank under the accession no. AWSQ00000000. The version described in this paper is version AWSQ01000000.
